# 59461 Underutilization of Program Services: Patterns, Sources, and Proposed Solutions

**DOI:** 10.1017/cts.2021.585

**Published:** 2021-03-30

**Authors:** Julie T. Elworth, Maon Billaud, Melissa Vaught, Jillian Harvey, Rechelle Paranal, Adrienne Zell, Charbel El Bcheraoui

**Affiliations:** 1 University of Washington; 2 Medical University of South Carolina; 3 Oregon Clinical and Translational Science Institute; 4 Robert Koch Institute, Berlin, Germany

## Abstract

ABSTRACT IMPACT: Investigators in clinical and translational research will be better supported by CTSAs that reduce barriers to the efficient and effective utilization of their program services. OBJECTIVES/GOALS: Evidence from three CTSAs show that investigators do not take advantage of all program services provided. This session will explore the patterns and sources of underutilization by sharing preliminary results from investigators and program managers. METHODS/STUDY POPULATION: Interviews with investigators at all three CTSA sites were conducted in Spring 2020. Investigators who had only used one program service and those who had used multiple program services across a span of three-years (2016 to 2018) were included in the sample. Investigators who had only used REDCap, were excluded. Interviews numbered about six interviews per site. Content analysis helped identify emerging themes and patterns. A survey with program managers at the three CTSA sites will be deployed in January 2021. Program managers across all programs will be included in the sample. Basic descriptive analysis will be used to analyze the data. RESULTS/ANTICIPATED RESULTS: Interviews with investigators at all sites illuminated ways different investigators use, learned about and leveraged services, as well as the barriers they encountered to using cross-program services. Investigators also provided thoughts on ways to address lack of program service use. Survey results are expected to clarify program managers’ knowledge of other program services in their CTSA, programs’ referral processes, and the barriers program managers face when recommending other services to users. DISCUSSION/SIGNIFICANCE OF FINDINGS: When investigators do not take full advantage of all program services provided, investigators are put at risk of having longer, less successful projects. Knowing the sources of underutilization can help curb this trend and effectively address investigator needs throughout the research process.

